# Ethical challenges in genetic research among Philippine Indigenous Peoples: Insights from fieldwork in Zamboanga and the Sulu Archipelago

**DOI:** 10.3389/fgene.2022.901515

**Published:** 2022-10-13

**Authors:** Jae Joseph Russell B. Rodriguez, John Meldwin D. Cuales, Michael James B. Herrera, Louward Allen M. Zubiri, Richard N. Muallil, Altan I. Ishmael, Edlyn B. Jimenez, Mark Stoneking, Maria Corazon A. De Ungria

**Affiliations:** ^1^ DNA Analysis Laboratory, Natural Sciences Research Institute, College of Science, University of the Philippines Diliman, Quezon City, Philippines; ^2^ Genetic and Molecular Biology Division, Institute of Biological Sciences, College of Arts and Sciences, University of the Philippines Los Baños, Laguna, Philippines; ^3^ Department of Evolutionary Genetics, Max Planck Institute for Evolutionary Anthropology, Leipzig, Germany; ^4^ Archaeological Studies Program, University of the Philippines Diliman, Quezon City, Philippines; ^5^ Independent Researcher, Malabon City, Philippines; ^6^ Office of Continuing Education and Extension Services, Mindanao State University—Tawi-Tawi College of Technology and Oceanography, Tawi-Tawi, Philippines; ^7^ Sama Studies Center, Mindanao State University—Tawi-Tawi College of Technology and Oceanography, Tawi-Tawi, Philippines; ^8^ National Institutes of Health, University of the Philippines Manila, Manila City, Philippines; ^9^ Universite Lyon 1, CNRS, Laboratoire de Biometrie et Biologie Evolutive, Villeurbanne, France; ^10^ Program on Biodiversity, Ethnicity, and Forensics, Philippine Genome Center, University of the Philippines, Quezon City, Philippines

**Keywords:** population genetics, Philippine Indigenous peoples, research ethics, Zamboanga, Sulu Archipelago, Sama, Tausug

## Abstract

The Philippines, with the recent discovery of an archaic hominin in Luzon and an extensive ethnolinguistic diversity of more than 100 Indigenous peoples, is crucial to understanding human evolution and population history in Island Southeast Asia. Advances in DNA sequencing technologies enable the rapid generation of genomic data to robustly address questions about origins, relatedness, and population movements. With the increased genetic sampling in the country, especially by international scientists, it is vital to revisit ethical rules and guidelines relevant to conducting research among Indigenous peoples. Our team led fieldwork expeditions between 2019 and February 2020 in Zamboanga and the Sulu Archipelago, a chain of islands connecting the Mindanao and Borneo landmasses. The trips concluded with a collection of 2,149 DNA samples from 104 field sites. We present our fieldwork experience among the mostly sea-oriented Sama-Bajaw and Tausug-speaking communities and propose recommendations to address the ethical challenges of conducting such research. This work contributes toward building an enabling research environment in the Philippines that respects the rights and autonomy of Indigenous peoples, who are the rightful owners of their DNA and all genetic information contained therein.

## 1 Introduction

The Philippines, an archipelagic nation in Island Southeast Asia, has figured in at least two important migration events in the region, namely the human settlement of Sunda and Sahul about 40,000 years ago (YA) (O' Connell and Allen, 2004) and the spread of Austronesian speaking farmers from Taiwan around 4,000–5,000 YA (Blust, 1995; Bellwood, 1997; Gray et al., 2009). Today the country is inhabited by more than 100 ethnolinguistic groups exhibiting cultural and phenotypic diversity (United Nations, 2007). The so-called “Negrito” phenotype possessed by certain groups (Barrows, 1910) and a “sea-nomadic” lifestyle adopted by some coastal communities (Nimmo, 2001) are of particular anthropological interest. These unique populations, along with the discovery of a new hominin species, *Homo luzonensis,* in the largest Philippine island of Luzon (Détroit et al., 2019), increasingly generate attention from scholars interested in how human evolution and prehistory unfolded in this part of the globe.

**FIGURE 1:**
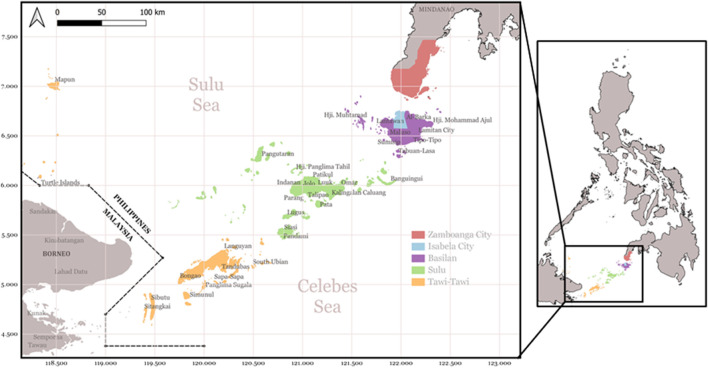
The Sulu Archipelago and Zamboanga City, Philippines. The map was created using QGIS version 3.22.2. A free and open-source Geographic Information System project by Open-Source Geospatial Foundation (OSGeo). http://qgis.osgeo.org.

Of notable historical, linguistic, and anthropological importance is the Sulu Archipelago, a chain of islands stretching in a northeast-southwest direction between the Zamboanga peninsula of Mindanao Island in the Philippines and northeast Borneo (Figure 1). This area is divided into the three provinces of Basilan, Sulu, and Tawi-Tawi, which are part of the Bangsamoro Autonomous Region in Muslim Mindanao (BARMM; see list of abbreviations and acronyms used in this article). Isabela City in Basilan Island and Zamboanga City at the southwestern extremity of Mindanao Island belong to Region IX, one of the country’s 16 administrative regions. Jolo Island, near the center, was the seat of the Sultanate of Sulu, a dominant maritime power that emerged 600 years ago following the introduction of Islam (Warren, 1985). Jolo is considered the homeland of the Tausug people, whose diaspora has spread to most parts of the archipelago. Seven Indigenous languages are spoken in the island chain as defined in the Ethnologue (Eberhard et al., 2021): Yakan, Sama Bangingi, Sama Pangutaran, Central Sama, Southern Sama, Jama Mapun, and Tausug^1^. Except for Tausug, these languages are grouped as Sama-Bajaw and are classified under Greater Barito languages (Pallesen, 1985; Blust, 2007). Although the Tausug language is currently the region’s lingua franca, the islands are populated by economically diverse groups of Sama speakers whose dialects are associated with certain villages, islands, or municipalities (Sather, 1997; Nimmo, 2001). The Yakan and Jama Mapun reside in Basilan and Mapun Islands, respectively, both groups having unmistakable cultural and linguistic connections to the Sama. The Sama Dilaut, more commonly known as the Badjao, live in coastal stilt houses throughout the region. As former boat-dwellers, they are often described as “sea nomads” or “sea gypsies” (Sather, 1997; Nimmo, 2001; Bellina et al., 2021).

DNA is a powerful tool for investigating questions about origins, prehistoric movements, and biological affinities. Earlier genetic studies of Philippine groups focused on uniparental lineages, shedding light on maternal and paternal histories (Tabbada et al., 2010; Delfin et al., 2011; Gunnarsdóttir et al., 2011; Delfin et al., 2014). With the development of single nucleotide polymorphism (SNP) microarrays and advanced sequencing technologies, genome-wide and high-coverage whole-genome sequence data promise more robust investigations of past demographic events and genetic adaptations (Reich et al., 2011; Lipson et al., 2014; Pagani et al., 2016; Skoglund et al., 2016; Jinam et al., 2017, GenomeAsia100K Consortium, 2019). Recent genetic studies spanning the entire country put forward far-reaching generalizations about the number, origins, and timeframe of dispersals into the Philippines (Larena et al., 2021a) and levels of archaic introgression from Denisovans (Larena et al., 2021b). Questions were raised concerning the ethical compliance of the researchers (National Commission on Indigenous Peoples, 2015; National Commission on Indigenous Peoples, 2021a; Philippine Genome Center, 2021; Rochmyaningsih, 2022). The long-term social ramifications of such alleged violations of ethical compliance are yet to be discerned. Nonetheless, past genetic studies lacking a sound ethical framework have resulted in several social harms, such as violation of individual rights, stigmatization, and general distrust of genomic research by Indigenous peoples (TallBear, 2007; Sterling, 2011; Chennells and Steenkamp, 2018). Given the growing interest from international researchers keen to study Island Southeast Asia, there is an urgent need to revisit Philippine policies, formulate clear guidelines where gaps remain, and require the compliance of all stakeholders to recognize and protect the rights of Indigenous peoples.

From 2019 to February 2020, our team conducted sampling campaigns in local communities in Zamboanga City and the Sulu Archipelago to investigate population genetic history and adaptations to a marine-oriented lifestyle. In this paper, we describe our best field practices and provide an overview of the conduct of genetic research among Philippine Indigenous peoples in compliance with existing national regulations. We intend this to be a helpful resource in reviewing research and ethical policies governing human genetics research in the Philippines.

## 2 Ethical guidelines for human genetic research in the Philippines

**FIGURE 2:**
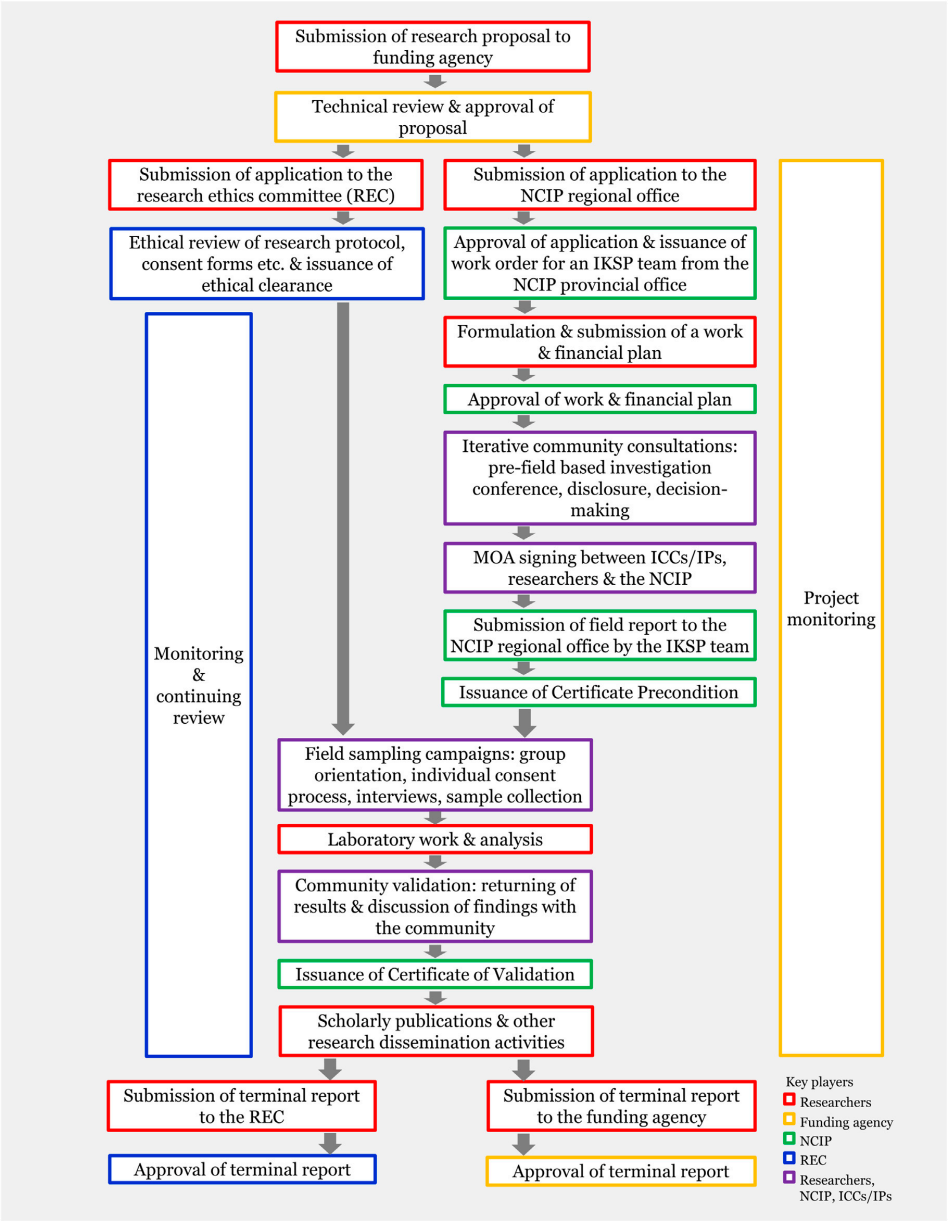
Stages of basic academic research among Philippine Indigenous cultural communities/Indigenous peoples (ICCs/IPs).

Human research in the Philippines requires compliance with national ethical rules and guidelines (Figure 2). The Republic Act No. 10532, or the Philippine National Health Research System Act of 2013, mandates that all health and health-related research should adhere to the guidelines of the Philippine Health Research Ethics Board (PHREB) (Philippine National Health Research System, 2013). PHREB adopts the WHO definition of health which is “a state of complete physical, mental, and social well-being and not merely the absence of disease or infirmity” (World Health Organization, 2006, p.1). Thus, studies involving human subjects conducted in the country, including human population genetic research, are within its purview, even if not necessarily medical in scope. They must undergo “must undergo ethical review and clearance before implementation to ensure the safety, dignity, and well-being of research participants” (Department of Science and Technology, 2012, p.1). Such reviews can only be conducted by research ethics committees (RECs) accredited by PHREB.

The involvement of Indigenous peoples in research adds another level of review and monitoring by institutions mandated to protect their rights and interests, primarily the National Commission on Indigenous Peoples (NCIP), established through Republic Act 8371 or the Indigenous Peoples Rights Act of 1997. The NCIP defines Indigenous peoples: as “indigenous on account of their descent from the populations which inhabited the country, at the time of conquest or colonization, or at the time of inroads of non-indigenous religions and cultures, or the establishment of present state boundaries, who retain some or all of their own social, economic, cultural and political institutions” (National Commission on Indigenous Peoples, 1997, p.3). The NCIP has published a list of Indigenous peoples it recognizes (National Commission on Indigenous Peoples, 2021b). Within BARMM, the Ministry of Indigenous Peoples Affairs (MIPA) performs similar functions and superseded the Office for Southern Cultural Communities (OSCC) after the promulgation of the Bangsamoro Organic Law in 2019.

The NCIP released two administrative orders in 2012. NCIP Administrative Order No. 1, known as “The Indigenous Knowledge Systems and Practices (IKSPs) and Customary Laws (CLs) Research and Documentation Guidelines of 2012” provides guidelines for academic research and community-initiated studies that could be used for policy formulations and/or implementation of NCIP mandates (National Commission on Indigenous Peoples, 2012a). NCIP Administrative Order No. 3 or “The Revised Guidelines on Free and Prior Informed Consent (FPIC) and related processes” applies to field-based investigations needed to ensure the protection of the rights “to ancestral domains, social justice, and human rights, self-governance and empowerment, and cultural integrity” (p.1) in projects aimed at commercializing Indigenous products and knowledge and those that could affect ancestral domains of the communities (National Commission on Indigenous Peoples, 2012b). In human genetic research, there is a need to distinguish basic academic research from studies with potential commercial gain (e.g., drug discovery), given the uncertainties in understanding bioprospecting, data and sample ownership, and the wealth of new information contained in individual genomes. Both Administrative Orders require forming an IKSP team, approving field plans, conducting iterative community consultations, and signing a Memorandum of Agreement (MoA) between the Indigenous groups, the researchers, and the NCIP. The MoA requires the researchers to return to the field sites, discuss the research findings, and consult the communities before publishing the study results. This is known as the research validation phase, which ensures the participants are among the first to learn about the results of the study.

The requirement for compliance with the NCIP AOs by researchers involved in studies with Indigenous groups was upheld in a Memorandum of Understanding (MoU) between NCIP and PHREB (Philippine Health Research Ethics Board, 2016), which aimed to reinforce each agency’s mandate. Under this MoU, researchers must obtain clearances from the NCIP and a PHREB-accredited Level 2 REC for all studies involving Indigenous peoples. This cooperation resulted in the requirement for research to include 1) an iterative and documented process of community consultations; 2) the use of informed consent forms that are understandable to all participants and preferably translated into the language of the Indigenous peoples; 3) biobanking and data sharing policies that include provisions for removal of samples/data; and 4) data privacy requirements following the Data Privacy Act of 2012.

PHREB also published the National Ethical Guidelines for Health and Health-Related Research (NEGHHR) in 2017 with a specific section on Indigenous peoples (Philippine Health Research Ethics Board, 2017). This section reiterates the requirement to obtain a clearance from the NCIP and discusses pertinent issues such as cultural sensitivity, vulnerability, and benefit-sharing and ownership. The NEGHHR also has a provision for the transfer of custody of biological samples to foreign institutions, which should follow a Material Transfer Agreement (MTA) that complies with all applicable international and Philippine regulations. The MTA must define the responsibilities of foreign researchers, including identifying a local counterpart researcher following the CIOMS guideline on collaborative partnership and capacity-building for research (Council for International Organizations of Medical Sciences, 2016).

## 3 Methodology: Regulatory compliance, social preparations, and fieldwork

This human genetic research with linguistics and animal archaeogenetic components comprises an interdisciplinary project investigating the history and adaptations of the peoples of Zamboanga and the Sulu Archipelago. The proposal underwent technical review by the Philippine Commission on Higher Education and the Max Planck Institute for Evolutionary Anthropology as the Ph.D. thesis of JJRBR. Ethical approval to conduct the study was granted by the University of the Philippines Manila Research Ethics Board (UPMREB 2018-453-01) and the Ethics Council of the Max Planck Society (Application No: 2021-22). UPMREB continuously monitors the project implementation and reviews amendments to the approved protocol.

Following NCIP AO No. 1, the fieldworkers and the IKSP team organized iterative disclosure processes, decision-making, and MoA signing with Sama/Sama Dilaut and Yakan communities in Basilan and Zamboanga City. Upon reviewing the field report, the NCIP Region IX office issued the Certificate Precondition, which officially signifies the full compliance of the researchers to NCIP requirements. We also applied for and received similar clearances from the OSCC in Tawi-Tawi and Sulu. While the Tausug is not among the Indigenous groups recognized by the NCIP or the OSCC, we nonetheless conducted consultations at the village level and committed to a similar validation process, as we recognized the value of following similar procedures for all communities in the study. On all field trips, the team conducted community activities in the presence of NCIP or OSCC representatives.

A collaborative partnership was formalized with the Mindanao State University–Tawi-Tawi College of Technology and Oceanography (MSU-TCTO), which serves as the local counterpart university in Tawi-Tawi. The MSU-TCTO researchers assisted the research team in the field and translated consent forms and agreements into Sama and Tausug.

Because the region is recognized as a site of violent conflict (International Alert, 2020), security precautions were observed by requesting the police to accompany the research team to some sites, particularly in the Sulu province. The police officers were primarily local Tausug or Sama and were often members of the communities. In our impression, they did not influence the consent process, and their presence indicated peace and order in the vicinity. With the local government divided into provinces, cities, municipalities, and *barangays* (small administrative districts corresponding to villages) the team approached governors, mayors, chairpersons, or their representatives during each courtesy call, where the research was explained, and assistance in reaching the communities was sought. On-site, the team consulted with the village leaders before meeting with the locals.

**FIGURE 3:**
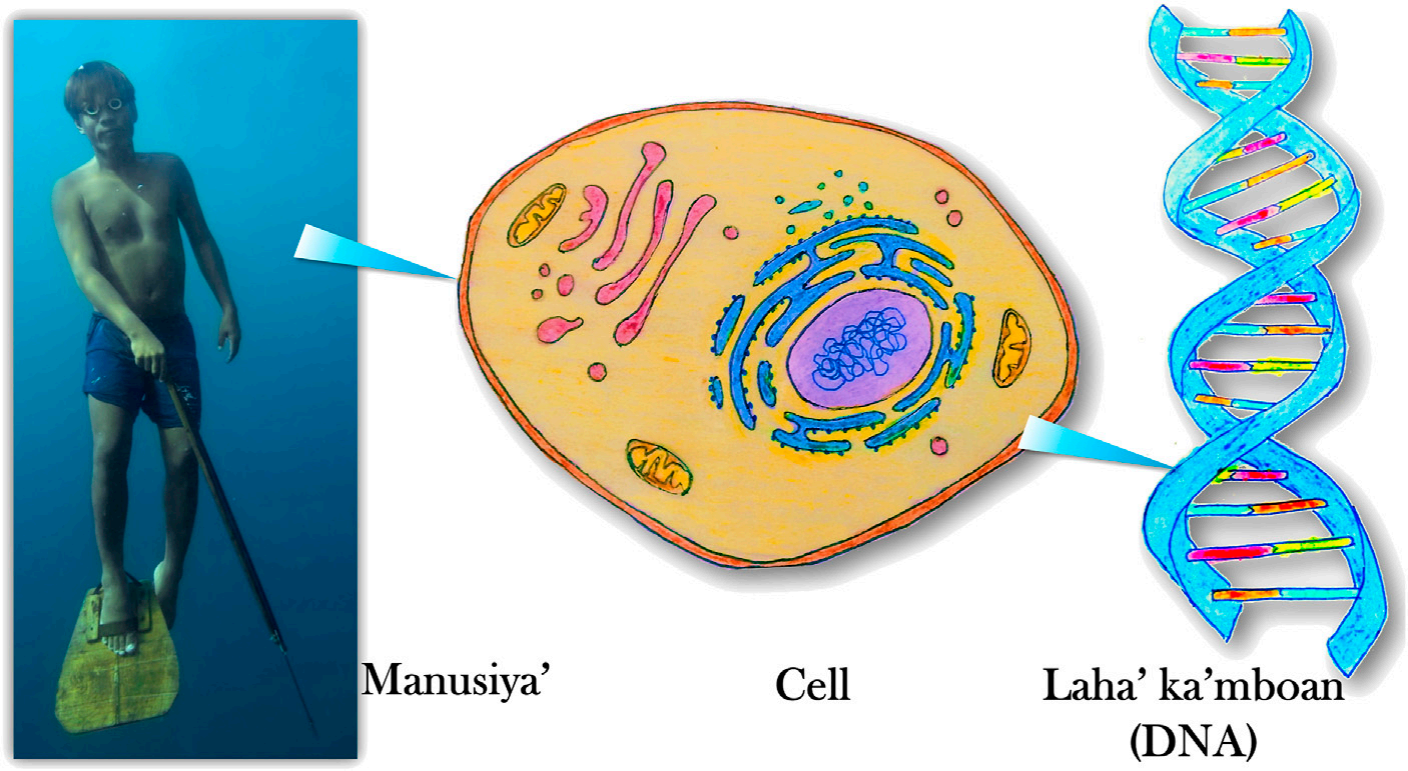
One of the visual aids used during group orientations explains how humans (*manusiya’*) are made up of cells containing DNA (*laha’ ka’mboan* lit. Blood of ancestors). Illustrated by Jacob Barbosa. Photograph from Jacob Maentz.

Sample collections proceeded in communities that signified consent. Fieldworkers discussed the study objectives and procedures with prospective participants in the local language using visual aids (Figure 3) with assistance from locals who were native speakers of Sama or Tausug. Individual consent was signified by signing or placing thumbprints on approved forms for participation in this study, for potential secondary use of samples, and a 15-year provision for sample storage. Donors were 18 years or older, except for some children who were part of family trios. Minors completed the assent forms that accompanied the parental consent forms. The field team also collected participants’ age, birthplace, group affinity, birthplace, and group affinity for their parents and grandparents (if known), diet, lifestyle, oral health practices, height, and weight. During the interview, the research team reconstructed the pedigrees of a participant’s immediate and extended relatives. Finally, a 2 ml saliva sample was provided by each volunteer.

The research team conducted five fieldwork expeditions from 2019 to February 2020, which involved multiple trips across islands on sea vessels. It culminated in a collection of 2,149 DNA samples from 104 villages, the most extensive set of samples from any Philippine region. At the time of this writing, sample processing and data analysis are ongoing simultaneously at the Max Planck Institute for Evolutionary Anthropology, Leipzig, Germany, and the DNA Analysis Laboratory and the Philippine Genome Center at the University of the Philippines Diliman.

## 4 Challenges and recommendations

### 4.1 Difficulties in communicating the value of genetic research to the communities

Communication at a basic level was a challenge from the outset. Despite being part of the Philippines, the Sulu Archipelago has a degree of distinction from the rest of the country, given its history of staunch resistance to colonial rule, a Muslim majority, and a long-standing clamor for autonomy or separation from the Philippine state. In 2019, the Bangsamoro Organic Law was ratified, formally installing a regional parliamentary government within the unitary and presidential national government (Official Gazette, 2018). The populations of the Sulu Archipelago predominantly speak languages other than the national language (Filipino), namely Sama and Tausug. Particularly in Sama Dilaut communities, many individuals had not received formal schooling and thus were not able to read and converse in Filipino.

To facilitate fieldwork, JJRBR and JMDC took on the challenge of learning to converse in the Sama language. Assisted by local partners, they explained the study, responded to questions, and conducted the interviews during community consultations in 104 field sites. During conversations in the field, it was evident that the concept of DNA was foreign to the worldview of many locals. However, they could grasp the concepts of ancestry and inheritance. Blood is viewed as a carrier of biological traits in the Filipino psyche, i.e., the gene (Tan, 2008). The idiom “it is in the blood” indicates that certain characteristics run in the family. One co-author, A.I.I. coined the term *laha’ ka’mboan* meaning “blood of the ancestors” as an equivalent term for DNA. In doing so, the DNA was likened to a bond that connects someone alive to one’s ancestors, and by extension, to others to whom they may be related “by blood”. That such an entity is present in all body parts made the participants appreciate why their saliva samples had to be collected. This demonstrates how scientists can communicate their research better by translating technical questions into more relatable and profound inquiries humans have always asked: where we come from and how we are related to one another.

### 4.2 Potential misconceptions and inconsistencies between genetic results with indigenous peoples’ oral histories

Communicating research findings accurately but in culturally acceptable ways will be challenging for the next phase of the study. Prior to publication, genetic results must undergo an output validation. The research team must return to inform and co-interpret the study findings with the communities (Figure 2). Gaps in understanding the limits of genomic inference occasionally raised inquiries on whether DNA can be used to test one’s group membership, like the “blood quantum” concept (McKay, 2021), or determine one’s descent from a prominent historical figure. Moreover, potential inconsistencies between genetic findings and folk narratives may prompt disagreeing sentiments. Previous research in other disciplines is instructive in this regard. For example, historical linguistics postulated the timing of the arrival of the Tausug in Jolo (Pallesen, 1985), which countered some local perceptions of autochthony in the region.

To avoid confusion when explaining the genetic findings, the team included researchers who teach in MSU-TCTO and belong to the Sama and Tausug communities. They are involved in discussions during data generation and analysis. In addition, the preparation for the validation phase will involve more local partners from MSU-TCTO, NCIP, the former OSCC, and MIPA. Field materials will be consulted with community leaders before they are used to resolve possible disputes with oral histories while maintaining the scientific integrity of the findings.

### 4.3 Defining different populations and the need for researchers to understand the cultural/historical basis for these groupings

In common practice, fieldwork in population genetic research involves recruiting participants of self-reported unadmixed ancestry who can at least ascribe that their four grandparents belong to the same ethnic group or reside in the general location. The availability of distinct groupings is desirable as most genetic analyses require that data be grouped into discrete samples representing defined ancestry groups, geographic regions, or languages of interest. However, in this study, grouping individuals into discrete clusters is challenging due to two factors: 1) the continuous nature of biological diversity and 2) the complexities of ethnonyms in the Sulu archipelago.

The arbitrariness of population boundaries becomes more apparent with increasing evidence that humans have always moved about and intermixed. Previous studies reported that Filipino groups were descended from Austronesian-speaking farmers who mixed with established local hunter-gatherers in the Philippine archipelago (Lipson et al., 2014; Jinam et al., 2017). Quasi-racial categorizations akin to those applied to continental groups (Lewis et al., 2021) are even more inappropriate when applied to very localized geographic regions, as intermarriages and migrations between islands potentially blur any semblance of genetic divides. Given this background, it will not be surprising to find genetic evidence for the mixing of Sama and Tausug ancestors and other populations integrating into the melting pot that was the Sultanate of Sulu.

Extensive analysis of historical and anthropological literature and interactions with locals proved extremely helpful in understanding the contexts of group identities and ethnonyms. From the history of the last centuries, episodes of violence and political changes have influenced human migration in the Sulu Archipelago (Warren, 1985; Nimmo, 2001), and the sea has been an avenue of migration and interactions between islands. Notably, a few villages in Tawi-Tawi were founded by Tausug or Bangingi settlers from Sulu who mixed with the neighboring populace, adopted the Sama dialect of the vicinity, and currently identify as the local Sama. Tausug and Sama intermarriages are common, resulting in children who may identify as both. It is also not uncommon for an individual to have been born in a site different from where the field collection was conducted or have one’s grandparents originating from other islands.

Ethnonyms or group names in the Sulu archipelago are multi-layered, with a few eliciting pejorative connotations. For example, “Sama” is a broad term many participants identify with when asked about group affiliation. However, the Ethnologue distinguishes between Central Sama and Southern Sama languages, broadly corresponding to the geographic distribution of dialects spoken across Sulu and Tawi-Tawi (Eberhard et al., 2021). In the field, locals do not consciously adhere to linguistic classification but would instead use an island name (e.g., *Sama deya*, lit. Sama on land, *Sama bihing* lit. Sama onshore) to specify their group identity. Likewise, the Tausug (lit. People of Sulu) may distinguish between *Tau gimba* (forest people) or *Tau higad* (shore people) or refer to names of municipalities (e.g., *Tau Maimbung, Tau Parang*).

*Sama Dilaut* is an example of an ethnonym with various connotations. It is the name former boat dwellers would use to refer to themselves, meaning “Sama of the sea”. The perceived eccentricity of “sea nomadism” practiced by the *Sama Dilaut*, their adherence to animistic beliefs, and their reluctance to integrate into mainstream society have contributed to their long history of social isolation and ostracism. Pejorative labels (e.g., *Luwaan* lit. spitted-out) were also sometimes applied to them (Nimmo, 2001). The *Sama Dilaut* is more commonly known by the exonym, Badjao (or Bajau), and many of them throughout the Philippines have adopted this name rather neutrally. However, researchers need to be aware of the various attitudes of other Sama (non-Sama Dilaut) communities towards being identified with the term. The attitudes range from its acceptance as being synonymous with Sama (especially in nearby Malaysia) to being appropriate only to sea nomads.

Moreover, a person’s ethnic identity may sometimes have little to do with ancestry. In Mapun, many locals who descended from Tausug ancestors, having spent their entire lives on the island and primarily speaking Jama Mapun, identify as Jama Mapun. JJRBR and JMDC later learned that “Jama” means “person” and “Jama Mapun” means a person from Mapun.

Consequently, ethnic identities in this region are not as neatly grouped as would be convenient for data analysis. The multi-layered nature of terms and the fluidity by which individuals self-identify in Sama and Tausug communities suggest that ethnic labels are not perfectly congruent with biological affinities and must be viewed with the awareness of potential bias in using these terms to categorize data. Individuals with varying levels of admixture contributed samples to this dataset - from those whose grandparents reside on the same island, to individuals who describe themselves as broadly Sama in ancestry but whose ancestors originate from different islands, to those with multiple ethnicities in their family tree. Our approach is to explore ways data can be arbitrarily grouped and examine which sets more faithfully mirror genetic clusters. We further support the recommendation to use endonyms or self-applied group names in publications, or in cases when the complete consensus among the group is lacking, the use of neutral exonyms provided with explanatory notes (Max Planck Institute for Evolutionary Anthropology, 2021).

Upon returning to the field sites, the research team will work with local partners to increase the communities’ understanding of what science can and cannot explain. Researchers will discuss the limitations of genomic inquiry and emphasize relatedness, the multiplicity of ancestors, and movements and intermixing of peoples in the past and present. As the spread of languages or cultural features may not be accompanied by the spread of genes (Diamond and Bellwood, 2003), it will be emphasized that results are not expected to fit perfectly with written or oral history, nor does genetic ancestry confer identity. The information campaign will be done with Indigenous groups, local partners in MSU-TCTO, NCIP, OSCC, MIPA, and the general public.

### 4.4 Diverse sectoral appreciation of the Indigenous peoples’ ownership of their samples and associated data

The ownership of biological samples and the derived genetic information is an integral component of the informed consent of human participants in a research study. However, Indigenous peoples, many of whom are socially and economically vulnerable, are not familiar with the process and may have unknowingly provided broad consent for their samples to be used and stored in local and global databases. While data sharing, rapidly publishing results, and follow-up investigations are essential to advance the field, broad consent and unrestricted access to genomic data is counter to the Indigenous peoples’ autonomy over their genomic data (Garrison et al., 2019) and excludes communities from sharing in potential benefits (Tsosie et al., 2021). Even with individual anonymization, social harms arising from the unregulated use of genomic data cannot be precluded, given that such data are usually linked to information about group affiliation.

To protect the rights of persons and their communities, the United Nations issued the Declaration on the Rights of Indigenous Peoples in 2007, which stated that “Indigenous peoples have the right to maintain, control, protect and develop their cultural heritage, traditional knowledge and traditional cultural expressions, … including human and genetic resources” (p. 3). In this framework, individuals and their communities own their genomic data, which must be recognized by the researchers conducting the primary study, and in all subsequent research arising from further use of archived biological samples and the derived genetic data. Considering secondary data usage, a system employing controlled access sharing (Byrd et al., 2020), where an access committee evaluates whether the requesting party substantially deviates from the terms of the original consent (thus warranting new iterative consultations with the community), is desirable. Data access committees must include members from Indigenous communities or government agencies mandated to protect such groups. Local leaders or representatives should foster accountability from researchers and ensure that their ownership and autonomy over their genetic data are genuinely respected.

In this study, the individual and community ownership of samples and derived genetic data is recognized and documented in the MoA signed by the University of the Philippines, the NCIP, and the recognized leaders of each Indigenous group. Moreover, the protocol for handling, storing, and analyzing data was submitted to the UPMREB, which monitors the research team’s compliance with the approved protocol. This level of transparency in the use of samples and genetic information within the parameters of the consent provided clearly manifests researchers’ recognition of the Indigenous peoples’ ownership of their samples and genetic information.

### 4.5 Varying compliance with ethical requirements by researchers and other stakeholders

Ethical review aims to balance the need to protect human participants from possible harm with the conduct of research that is beneficial to the community and the general public. Ethical issues have been raised concerning scientists from high-income nations conducting research among Indigenous peoples from low- and middle-income countries (LIMC) (van Teijlingen and Simkhada, 2012; Lahey, 2013; Pasic et al., 2018; Schroeder et al., 2018). All too often, this involves “helicopter research”, i.e., research conducted under different ethical standards or with less oversight, with the source country having little say in the types of studies conducted, access to biological samples and study findings, and level of benefit sharing with the participants and their communities (Nature Journal, 2022).

As local or international scientists may choose not to follow regulations for protecting human participants, the responsibility for adhering to such rules must be shared by other stakeholders (De Ungria and Jimenez, 2022). For example, scientific journals are responsible for declining the publication of studies or retracting works where ethical misconduct has been demonstrated. Most journals uphold the protection of human participants as stipulated in the Declaration of Helsinki. However, this is primarily intended for physicians conducting patient research. As the scope of research involving humans extends to other disciplines beyond medical research, journals should consider upholding other relevant international ethical guidelines (De Ungria and Jimenez, 2021). In particular, the 2016 Declaration of Taipei (World Medical Association, 2016) and the CIOMS guidelines (Council for International Organizations of Medical Sciences, 2016) cover issues relating to biobanking, databases, and various aspects of human research. Journals should also require ethical compliance to globally accepted principles and legal guidelines in countries from which those genetic samples were sourced. In June 2022, Nature reported that it is improving its policies for publishing papers, following the recommendations made during the seventh World Conference on Research Integrity (Nature Journal, 2022). However, the scope of the policy review was unclear regarding the increased protection of the human participants, including Indigenous peoples, who in many LMICs are more socially and economically vulnerable than the general population.

Additionally, funding agencies are responsible for only supporting research that is ethical and investigating any allegations of misconduct by investigators of projects they finance. For example, the European Commission upholds the Global Code of Conduct for Research in Resource-Poor Settings (Schroeder et al., 2019) as a mandatory reference for the research and development activities it supports. Among other guidelines, the code admonishes researchers to seek ethical review in the host country, when available, even if an ethics approval has already been obtained in the researchers’ home country.

Lastly, research institutions have administrative oversight over their affiliated scientists and should ensure that laws and regulations are observed correctly in jurisdictions where they conducted their field studies. Such institutions should proactively conduct fair and thorough investigations whenever misconduct accusations arise. Some recent studies on Philippine Indigenous communities allegedly did not fully comply with local policies and proceeded to conduct research without the proper clearance from institutions accredited by PHREB (Rochmyaningsih, 2022). The highest level of accountability for this work is expected from scientists who continue to claim that they have the required ethics and institutional clearances, even after being informed that this was not the case (National Commission on Indigenous Peoples, 2015; National Commission on Indigenous Peoples, 2021a; Philippine Genome Center, 2021).

While it is the primary responsibility of researchers to ensure that the conduct of research is ethical, a research’s social and scientific value is best achieved through a multi-stakeholder cooperation. This includes research institutions, funding agencies, ethics review committees, government institutions, and scientific journals (De Ungria and Jimenez, 2022). This research is being conducted under the monitoring of the UPMREB, the NCIP, and OSCC/MIPA. Moreover, reports are submitted annually to the UPMREB to document our adherence to the approved protocol. This protocol underwent an ethics review before the start of the project. Any change or amendment to protocol also undergoes an ethics review process by the committee composed of social and natural scientists, lay people, and ethics experts before implementation to ensure the protection of the study participants.

During the fieldwork, research results were communicated in the local language. Forms and documents were translated, and scientific concepts were explained through visual aids. It was nonetheless evident in many instances that the community did not fully comprehend all scientific technicalities. In this study, the participant’s willingness to be part of a population genetics study relied heavily on the community’s trust in the researchers and our partners in MSU-TCTO, OSCC, and NCIP. A response often received roughly translated to: “For as long as it is for the common good, then I am willing to take part.” Valuing reciprocity in research means being duty-bound to maintain and honor the people’s trust during and after the study for as long as genomic data remains accessible for analysis and interpretation. Because of these field experiences where the communities shared not only their samples but also their trust with the research team, we highly recommend that scientists, particularly those who engage with Indigenous peoples aspire to set an example of good research practice in the community. The research must be characterized by transparency, honesty, accountability, and regulatory compliance as concrete manifestations of how scientists should value the trust afforded by Indigenous persons and their communities.

### 4.6 Need to provide direct social benefits to the research participants and their communities

In living in the Sama and Tausug communities, the research team became familiar with their pressing needs, including necessities such as access to healthcare, education, and means of livelihood. Poor understanding of Indigenous cultures, even by fellow Filipinos, remains a drawback, resulting in the misrepresentation of Indigenous peoples in government and the public domain. This lack of representation often manifests in governance that fails to prevent further displacement and loss of cultural identity and ancestral domains (Seitz, 1998; Human Rights Watch, 2014; Chandran, 2018).

As academic researchers, it is well beyond our capacity to implement systemic changes that ensure these needs are met. The social aspect of population genetic research and the value of interacting with communities beyond the normal boundaries of scientific research are too often overlooked especially with the intensifying race among scientists to publish novel findings. However, scientists must remember that maximizing beneficence and reducing the risk of social harm are fundamental principles of ethical research (World Medical Association, 2013). We, therefore, recommend that researchers find creative ways of helping the communities by tapping resources, particularly those from the educational and health sectors, and communicating through their extensive networks to connect agencies and people that could assist Indigenous peoples.

In addition, education and building research capacity are areas where communities can draw much social benefit (Claw et al., 2018). In some countries, scientists foster partnerships with Indigenous peoples through scientific training and internship programs with the long-term goal of equipping Indigenous scientists to carry-out genomic research to benefit their communities (e.g., Carl R, 2018). Efforts of this type of engagement were made in partnership with the MSU-TCTO. The research team delivered lectures on genetics, linguistics, and archaeology to students and the teaching staff of MSU-TCTO, many of whom are members of the Sama and Tausug communities. In seeking their assistance, the research team aims to understand how the Indigenous peoples view the results of this study using their cultural lens to design better information materials for the communities. Moreover, the research team has committed to crafting a university-level Sama studies course as part of the project in line with the CHED’s policies to integrate Indigenous people’s studies into the higher education curricula (Commission on Higher Education, 2019).

## 5 Conclusion

Genomic technologies applied to studies of present-day and ancient populations offer unprecedented insights into our history as a human species. Whereas it could be tempting to view national ethical procedures as bureaucratic obstacles, we believe that prioritizing the welfare of communities increases the research’s overall scientific and social value. While the field has rapidly advanced, Indigenous communities who are the focal subjects of such studies, remain vulnerable and marginalized, especially in low- and middle-income settings. To bridge this gap, it is crucial to recognize Indigenous peoples as partners in the research process, as rightful owners of genetic information, and place them on the receiving end of benefits derived from the study. Moreover, it cannot be overemphasized that this is a multi-stakeholder pursuit where academic institutions, government agencies, and journals play vital roles in ensuring that the highest ethical standards are upheld. Lastly, recognizing that every Indigenous group is unique, we encourage ethical, legal, and social implications studies to be conducted during community engagements. These would entail delving into local perceptions of communities regarding consent, traditional knowledge, ownership, and their needs and values, among others.

This paper aims to contribute knowledge to researchers, regulatory agencies, and policymakers by sharing our experiences and making recommendations to address our challenges during fieldwork. We call for revisiting existing regulations to enhance the protection of human participants, especially those who belong to Indigenous groups while creating an enabling research environment for the benefit of all stakeholders. Overall, these are essential steps toward improving the governance of ethical research in the Philippines and, possibly, in other countries undergoing similar challenges, generating research outcomes that are meaningful and respectful of the rights and needs of Indigenous peoples.

## Footnotes

1 We apply the names of ethnic groups to the names of the languages, consistent with the Ethnologue (Eberhard et al., 2021). Whereas these may deviate from the actual terms used by locals, they are, in our opinion, more neutral to the varying degrees of usage and preference by the locals. For example, there are at least two other names for the language spoken by the Tausug, namely *Bahasa Sūg* and *Sinug*. When “Tausug language” or “speaking Tausug” appears in the manuscript, it is a shorthand for “the language spoken by the Tausug people”.

## Abbreviations

AO, Administrative order; BARMM, Bangsamoro Autonomous Region in Muslim Mindanao; CIOMS, Council for International Organizations of Medical Sciences; IKSPs, Indigenous Knowledge Systems and Practices; MIPA, Ministry of Indigenous Peoples Affairs; MoA, Memorandum of Agreement; MoU, Memorandum of Understanding; MSU-TCTO, Mindanao State University—Tawi-Tawi College of Technology and Oceanography; MTA, Material Transfer Agreement; NCIP, National Commission on Indigenous Peoples; NEGHR, National Ethical Guidelines for Health and Health-Related Research; OSCC, Office for Southern Cultural Communities; PHREB, Philippine Health Research Ethics Board; RECs, Research Ethics Committees.
